# Effects of Different Warm-Up Strategies on Intermittent Performance and Physiological Responses in High-Level Water Polo Players

**DOI:** 10.3390/jfmk11030297

**Published:** 2026-07-28

**Authors:** João N. A. Dias, Daniel A. Marinho, Luís B. Faíl, Konstantinos Papadimitriou, Henrique P. Neiva

**Affiliations:** 1 Department of Sport Sciences, University of Beira Interior, 6201-001 Covilhã, Portugal; joao.nuno.alves.dias@ubi.pt (J.N.A.D.); marinho.d@gmail.com (D.A.M.); luis.brandao.fail@ubi.pt (L.B.F.); 2 Research Center in Sports Sciences, Health Sciences and Human Development, CIDESD, 6201-001 Covilhã, Portugal; 3 Department of Nutritional Science and Dietetics, International Hellenic University, Sindos, 57400 Thessaloniki, Greece; 4 Faculty of Sports Sciences and Physical Education, Metropolitan College, Thessaloniki Campus, 54625 Thessaloniki, Greece

**Keywords:** pre-exercise, aquatic sports, specificity, intensity, lactate, heart rate

## Abstract

**Objectives**: This study aimed to examine the effects of three different warm-up protocols on performance, physiological, and perceptual responses in high-level water polo players. **Methods**: Ten international-level athletes (Tier 4; mean ± SD: age 22.4 ± 3.53 years; height 1.78 ± 0.06 m; body mass 75.83 ± 6.44 kg) completed three experimental conditions in a randomized, counterbalanced design: dryland, in-water, and combined warm-up. Following each warm-up condition, participants performed the Water Polo Intermittent Shuttle Test (WIST), while performance (distance covered and average speed), blood lactate concentration ([La^−^]), heart rate (HR), tympanic temperature, and rating of perceived exertion (RPE) were assessed. Statistical significance was set at *p* ≤ 0.05. **Results**: Players covered greater distances following the in-water (331.5 ± 151.24 m) and combined (358.5 ± 134.06 m) warm-ups compared to dryland (226.5 ± 129.51 m; *p* < 0.01; ES = 2.00 and 1.53, respectively), with no significant differences between in-water and combined conditions (*p* = 0.381; ES = 0.29). Physiological responses following the WIST were largely similar between conditions, although [La^−^] was higher in the dryland condition (12.46 ± 4.46 mmol·L^−1^) compared to the combined condition at 10 min post-exercise (8.66 ± 2.47 mmol·L^−1^; *p* = 0.03; ES = 0.80). No differences were found for HR, tympanic temperature, or RPE. **Conclusions**: These findings suggest that warm-up protocols including an in-water component are more effective than dryland-only routines for optimizing intermittent performance in water polo players. However, combining dryland and in-water exercises does not appear to provide additional benefits compared to in-water warm-up alone.

## 1. Introduction

Warm-up is commonly performed to enhance physiological and psychological readiness, thereby optimizing subsequent exercise performance [1,2,3]. An effective warm-up elevates muscle and core temperature, which may improve metabolic efficiency, accelerate energy turnover, and facilitate force production during exercise performed at different intensities [4,5]. Additionally, warm–up strategies may also improve range of motion, strength, and movement coordination, and prepare the cardiovascular and neuromuscular systems for the demands of the subsequent task [6,7,8]. In aquatic sports, however, findings regarding the effectiveness of different warm-up strategies remain mixed [7,9,10,11], suggesting that their benefits depend on both the characteristics of the protocol and the demands of the activity performed thereafter [7,9,12]. This may be particularly relevant in intermittent sports, which require repeated high-intensity efforts interspersed with recovery periods and the rapid contribution of both aerobic and anaerobic energy systems [13]. Evidence from basketball [14], futsal [15], soccer [16,17,18], and handball [19,20] demonstrates that appropriate warm-up routines can improve sprint performance, power output, and agility, and, in some contexts, contribute to injury-risk reduction. These findings suggest that an effective warm-up should provide sufficient physiological and neuromuscular activation while also reflecting the specific movement and energetic demands of the subsequent activity.

Water polo is a high-intensity intermittent sport that requires rapid transitions between maximal efforts, defensive actions, and active recovery [21,22]. It also involves specific technical and physiological demands, such as head-up swimming, vertical eggbeater kicking, upper-limb explosive actions, and both anaerobic and aerobic contributions, respectively [23,24,25]. Additionally, the aquatic environment imposes unique constraints such as buoyancy and drag forces, which influence body position, propulsion, movement efficiency, and energy expenditure [26]. This way, the effectiveness of a warm-up in water polo may depend on the physiological activation achieved but also on whether the protocol prepares athletes for the specific coordinative and mechanical demands of movement in water.

In-water warm-ups are commonly used to increase body temperature, improve stroke efficiency, and activate neuromuscular patterns that closely resemble competition [8,27]. However, dryland warm-up routines, frequently used before entering the water, may enhance muscle activation, joint mobility, and neuromuscular recruitment through dynamic and resistance-based exercises [6,9]. From an applied perspective, coaches frequently combine dryland and in-water routines, although evidence directly comparing their effectiveness remains limited [28]. The interval between the end of the warm-up and the start of competition represents an additional practical consideration, particularly when access to the competition venue or pool is restricted [29]. Theoretically, combining dryland and in-water warm-up may provide complementary stimuli by integrating general activation with sport-specific aquatic preparation [30]. Nevertheless, it remains unclear whether this combination provides additional benefits compared with an adequately structured in-water warm-up alone. Moreover, because the benefits of warm-up may progressively decline during passive recovery, the transition period should be considered and standardized when comparing different warm-up strategies [12,31].

Despite the practical relevance of warm-up for aquatic sports, most studies have primarily focused on swimming performance, particularly time-trial outcomes, rather than intermittent protocols that more closely reflect the demands of water polo [30,32,33,34]. This highlights the limited evidence regarding the effects of dryland, in-water, and combined warm-up strategies on ecologically relevant, sport-specific intermittent performance in water polo. Therefore, this study aims to examine the acute effects of three warm-up protocols, specifically, in-water warm-up, dryland warm-up, or combined warm-up, on performance outcomes in high-level water polo players. Additionally, physiological (heart rate: HR, and blood lactate concentration: [La^−^]) and perceptual (rating of perceived exertion, RPE) responses were evaluated. It was hypothesized that the combined warm-up would produce greater improvements in performance compared with the in-water and dryland warm-up alone.

## 2. Materials and Methods

### 2.1. Experimental Approach to the Problem

This study used a randomized, counterbalanced, within-subject repeated-measures design to examine the acute effects of three different warm-up protocols on intermittent performance and physiological responses in high-level water polo players. The three experimental conditions consisted of: (i) dryland warm-up, (ii) in-water warm-up, and (iii) combined dryland and in-water warm-up. The order of the experimental conditions was randomized and counterbalanced to minimize potential order effects. Each experimental condition was performed in a separate session at the same time of day, with at least 48 h between testing sessions to allow recovery from the preceding maximal Water Polo Intermittent Shuttle Test (WIST) and minimize the potential influence of residual fatigue and carryover effects [3,18]. This interval referred exclusively to the separation between experimental sessions. Participants maintained their regular training and competition schedules throughout the study but were instructed to avoid high-intensity exercise during the 24 h preceding each testing session.

The independent variable was the type of warm-up protocol, whereas the dependent variables included WIST performance (distance covered and average swimming speed), [La^−^], HR, tympanic temperature, and RPE. The WIST was selected because it reproduces the intermittent and sport-specific demands of water polo, including repeated head-up swimming efforts and changes in exercise intensity. All testing sessions were conducted under standardized environmental and temporal conditions, with participants completing each experimental condition in a randomized and counterbalanced order separated by at least 48 h. The researchers responsible for verifying the WIST were not involved in the administration of the warm-up protocol. The experimental design was intended to replicate practical competitive scenarios in which athletes perform different warm-up strategies before intermittent high-intensity exercise, thereby enhancing the ecological validity and practical applicability of the findings.

### 2.2. Subjects

All participants were familiar with the warm-up procedures used in this study, as similar routines are regularly implemented in both training and competitive contexts, thereby minimizing potential learning effects. Inclusion criteria required participants to be free from injury or physical limitations and to be actively involved in regular training and competition. Athletes who were injured, in rehabilitation, not training regularly, or not selected for competition were excluded. Before participation, all athletes completed a health and training history questionnaire that included information on anthropometric characteristics, training experience, and injury history.

Ten male water polo players competing in the Portuguese national league and who had represented the Portuguese national team participated in this study. Based on their competitive level and international representation, participants were classified as Tier 4 according to the Participant Classification Framework proposed by McKay et al. [35]. All eligible players from the participating team who were available during the data-collection period were recruited. The resulting sample size is comparable to those reported in previous studies involving water polo players [36,37,38,39]. Nevertheless, the relatively small sample should be considered when interpreting the precision and generalizability of the findings.

All athletes had at least six years of competitive experience and were regularly engaged in structured training and official competitions. Participants were aged 22.4 ± 3.53 years, with a body mass of 75.83 ± 6.44 kg and a height of 1.78 ± 0.06 m. They had a training experience of 10.9 ± 4.89 years and reported an average weekly training frequency of 4.1 ± 0.57 sessions, with each session lasting 2.06 ± 0.13 h. Participants were instructed to maintain their regular training schedule but to avoid high-intensity training during the 24 h preceding each testing session. They were also instructed to avoid caffeine and alcohol for at least 24 h before testing and to maintain similar dietary and hydration patterns across sessions to minimize potential confounding effects [40].

The study was approved by the Ethics Committee of the University of Beira Interior (CE-UBI-Pj-2025-035) and conducted in accordance with the Declaration of Helsinki. All participants provided informed consent before participation.

### 2.3. Procedures

**Figure 1:**
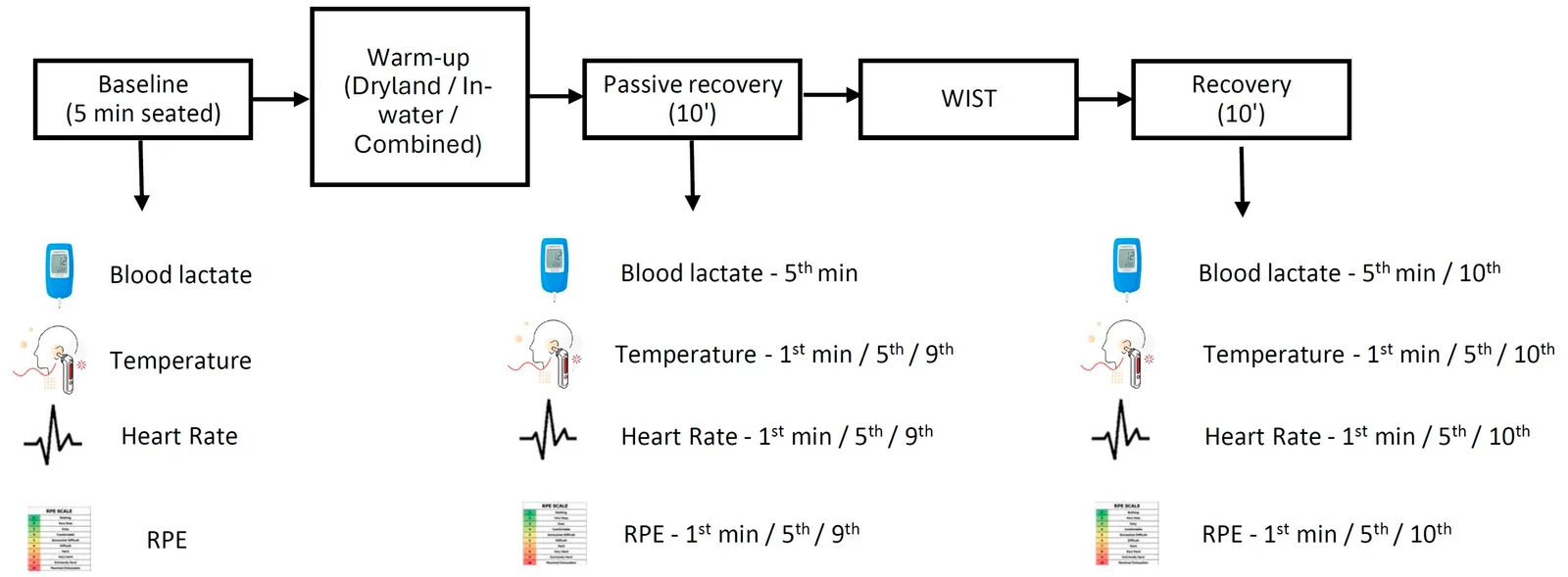
Schematic representation of experimental design. Participants completed three experimental conditions (dryland, in-water, and combined warm-up). Measurements of heart rate (HR), rating of perceived exertion (RPE), tympanic temperature, and blood lactate concentration ([La^−^]) were collected at baseline, after the warm-up, following a 5 min passive recovery period, and during recovery after the Water Polo Intermittent Shuttle Test (WIST) at 1, 5, and 10 min.

All experimental sessions followed a standardized experimental sequence (Figure 1) and were conducted during the final month of the competitive season, in a 25 m indoor swimming pool. Water temperature was maintained at 27.25 ± 0.35 °C and measured before each testing session to ensure consistency. All sessions were performed at the same time of day (afternoon) under standardized conditions. Upon arrival, participants confirmed compliance with the pre-test instructions. They were then seated for 5 min, with legs uncrossed and body relaxed, for baseline assessment of HR, rating of perceived exertion (RPE), tympanic temperature, and [La^−^].

After baseline assessment, participants performed the assigned warm-up protocol (dryland, in-water, or combined). The structure, duration, and specific exercises included in each warm-up protocol are presented in Table 1. Following the warm-up, participants remained seated for a 5 min passive recovery period, after which HR, RPE, tympanic temperature, and [La^−^] were measured again. Immediately thereafter, participants performed the Water Polo Intermittent Shuttle Test (WIST). After completion of the WIST, HR, RPE, and tympanic temperature were assessed at 1, 5, and 10 min post-exercise, whereas [La^−^] was measured at 5 and 10 min post-exercise. Standardized verbal encouragement was provided during all WIST trials.

Performance assessment. Performance was evaluated using the WIST, as previously described by Mujika et al. [30]. Players started from a stationary position at the lane rope and began swimming upon an audio signal. The test consisted of repeated shuttle efforts performed in a head-up swimming position between two lane ropes placed 7.5 m apart, reproducing a movement pattern specific to water polo. The initial four shuttles were performed at speeds ranging from 1.03 to 1.36 m·s^−1^, followed by seven shuttles at speeds between 1.43 and 1.46 m·s^−1^. Thereafter, speed increased by 0.005 m·s^−1^ every eight shuttles.

The test was terminated when a player could no longer maintain the required pace or failed to reach the lane rope on two occasions. The final level achieved was converted to total distance covered (m), which was considered the primary performance outcome. Average swimming speed during the test was also calculated. To ensure test validity and confirm that players reached the lane rope at each shuttle, two video cameras (Casio Exilim EX-F1, 30 Hz) were positioned to monitor performance [3]. Two experienced evaluators independently verified task completion during each shuttle.

Physiological and perceptual variables. [La^−^] was measured using a portable lactate analyzer (Lactate Pro2; Arkray, Kyoto, Japan), with capillary blood samples collected from the fingertip. Measurements were obtained at baseline, 5 min after each warm-up protocol, and at 5 and 10 min after the WIST.

Tympanic temperature was measured using a ThermoScan IRT 4520 thermometer (Braun, Kronberg, Germany). Tympanic temperature is considered a reliable indicator of core temperature and its response to exercise [41], with thermometers providing an accuracy of ±0.2 °C for temperatures ranging from 32 °C to 42 °C. HR was monitored using a Polar Vantage NV HR monitor (Polar, Kempele, Finland). At each assessment point, HR was expressed as the mean value recorded during the corresponding 1 min interval. Both variables were assessed at baseline, after the 5 min recovery period following the warm-up, and at 1, 5, and 10 min after the WIST. The RPE was assessed using the modified Borg 1-10 scale [42] at the same time points.

### 2.4. Statistical Analyses

Data are presented as mean ± standard deviation (SD) and 95% confidence intervals (CI). The normality of data distribution was verified using the Shapiro–Wilk test. A one-way repeated-measures analysis of variance (ANOVA) was used to examine the effects of the three warm-up conditions (dryland, in-water, and combined) on performance, physiological, and perceptual variables. Sphericity was verified using Mauchly’s test, and when violated, the Greenhouse–Geisser correction was applied. Post hoc pairwise comparisons were performed using the least significant difference (LSD) test, as this approach is appropriate in repeated-measures designs with a limited number of conditions and controlled within-subject variability. Absolute mean differences between conditions were also calculated for pairwise comparisons. Effect size was calculated to estimate variance between conditions (partial eta squared: ηp^2^), and Hedges’ g for within-subject comparisons (ES). For ηp^2^, cut-off values were interpreted as 0.01 for small, 0.09 for moderate, and 0.25 for large effects. For ES, values of 0.20, 0.50, and 0.80 were considered small, medium, and large, respectively. Statistical analyses were performed using IBM SPSS Statistics for Windows (version 29.0; IBM Corp., Armonk, NY, USA). The level of statistical significance was set at *p* ≤ 0.05.

## 3. Results

No significant differences were observed between the three warm-up conditions for any of the measured variables at baseline (all *p* > 0.05, Supplementary Table S1). A one-way repeated-measures ANOVA revealed a significant main effect of warm-up condition on WIST performance [F (2, 18) = 15.54; *p* = 0.003; ηp^2^ = 0.633], indicating a large effect. Post hoc pairwise comparisons showed that performance following the in-water condition (mean difference = 105 ± 52.4 m; *p* < 0.001) and the combined condition (mean difference = 132 ± 86.3 m; *p* = 0.009) was significantly higher than the dryland condition. No significant differences were observed between the in-water and combined conditions (*p* = 0.381). Similar results were observed for average swimming speed, with higher values in the in-water and combined conditions compared to dryland, and no differences between the in-water and combined conditions (Table 2 and Table 3).

**Figure 2:**
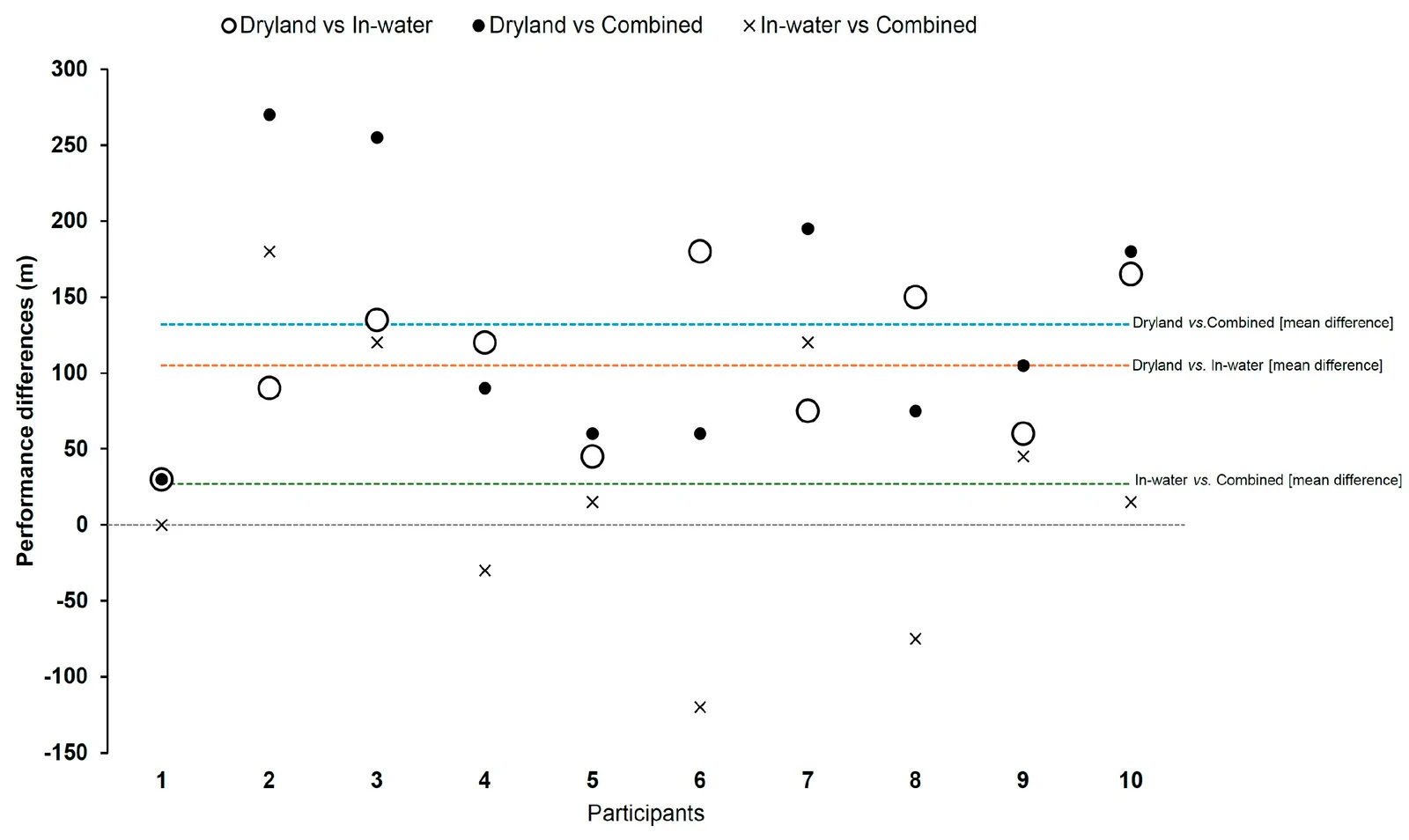
Individual differences in distance covered during the WIST across the three warm-up conditions (dryland vs. in-water, dryland vs. combined, and in-water vs. combined), including mean values and mean difference lines (n = 10).

Figure 2 illustrates the individual responses in WIST performance across the three warm-up conditions. When comparing the dryland and in-water conditions, all the participants showed a positive response, achieving greater distances following the in-water protocol. A similar pattern was observed when comparing the dryland and combined conditions, with all the participants improving performance following the combined protocol. In contrast, the comparison between the in-water and combined conditions showed a more heterogeneous response, with a similar number of participants demonstrating positive and negative responses.

Physiological and perceptual responses following the WIST are presented in Table 2 and Table 3. [La^−^] increased across all conditions. At 10 min post-exercise, [La^−^] was significantly higher in the dryland condition compared to the combined condition (*p* = 0.033), whereas no differences were observed between the remaining conditions. No significant differences were observed between conditions for HR at either 1 or 10 min post-exercise (all *p* > 0.05). Similarly, tympanic temperature did not differ significantly between conditions at either point (all *p* > 0.05). RPE values were also no different between conditions, with no significant differences observed at either 1 or 10 min post-exercise (all *p* > 0.05).

To provide additional context regarding the physiological state prior to the performance test, responses measured 5 min after the warm-up are presented in Table 4 and Table 5. Tympanic temperature was higher after the dryland warm-up compared to the in-water warm-up. HR was higher in the in-water condition compared to the combined condition. [La^−^] was higher in both the dryland and combined conditions compared to the in-water condition. No significant differences were observed for RPE.

## 4. Discussion

The present study examined the effects of three warm-up protocols on performance and physiological responses in high-level water polo players. The main finding was that both the in-water and combined warm-up protocols resulted in superior performance compared to the dryland condition, while no significant differences were observed between the in-water and combined protocols. These results highlight the importance of including a sport-specific, in-water component to optimize intermittent performance in water polo. This is consistent with previous literature demonstrating that warm-up strategies incorporating sport-specific movements are more effective in enhancing subsequent performance, as they promote greater neuromuscular readiness, coordination, and movement efficiency [22,43].

Contrary to the initial hypothesis, the combined warm-up did not significantly improve WIST performance compared with the in-water condition. Although the mean distance covered was numerically higher after the combined protocol, the between-condition effect was small, and individual responses were heterogeneous. A plausible explanation is that the in-water warm-up alone provided a sufficient task-specific priming stimulus [12,27,43]. Once this level of water-specific readiness had been achieved, the preceding dryland exercises may have provided limited additional preparation for the test. This interpretation is consistent with previous evidence indicating that combining general and specific warm-up activities does not necessarily provide greater performance benefits than a specific warm-up alone [6]. Moreover, the greater duration and physiological demand of the combined protocol may also have contributed to the lack of an additional performance benefit. The combined warm-up lasted approximately 40 min, whereas the in-water protocol lasted approximately 20 min, and post-warm-up [La^−^] was higher after the combined condition than after the in-water condition. This suggests that the additional dryland component increased the total exercise volume and metabolic demand without producing a proportional improvement in performance. It is therefore possible that any additional activation induced by the dryland exercises was compromised by the greater workload of the combined warm-up.

The superior performance observed after the protocols containing an in-water component may be explained by the specific demands of the WIST. The test reproduces several relevant demands of water polo, such as repeated head-up swimming over short distances, frequent acceleration and deceleration, and repeated changes in direction, which are not replicated during dryland-only routines. Performance depends not only on general physiological readiness, but also on the ability to rapidly establish an efficient horizontal body position, coordinate stroke timing, and apply propulsive force under the hydrodynamic constraints imposed by buoyancy and drag [44,45,46]. The in-water and combined warm-ups included head-up swimming, technical drills, eggbeater kicking, and water-polo-specific actions, which may have facilitated the neuromuscular coordination and movement organization required during the WIST. In contrast, although the dryland protocol may have increased general activation, it may not have adequately prepared athletes for the neuromuscular and technical demands of the WIST. Thus, the performance advantage observed after the protocols containing an in-water component may reflect a reduced technical and neuromuscular adjustment at the onset of the WIST [47,48], rather than a purely physiological effect. However, this interpretation should be considered cautiously because biomechanical and neuromuscular variables were not directly assessed.

The responses observed 5 min after the warm-up protocols may provide important context for the performance outcomes. At this stage, differences were found in [La^−^], HR, and tympanic temperature, indicating that each warm-up may have induced a distinct physiological state before the WIST. The dryland and combined conditions produced higher [La^−^] than the in-water condition; HR was higher after the in-water than after the combined warm-up, and tympanic temperature was higher after the dryland than after the in-water condition. Warm-up-induced changes in physiological state, including elevated oxygen uptake, increased muscle temperature, and enhanced enzymatic activity, are known to influence subsequent performance by possibly improving oxygen kinetics and reducing anaerobic contribution at exercise onset [12,44,48]. However, the fact that these differences were not maintained after the WIST suggests that the performance effect of warm-up may be more closely related to the transient physiological and neuromuscular state induced immediately before exercise rather than to post-exercise physiological responses. This is consistent with the concept of warm-up as a priming stimulus, whereby its primary effect is to enhance readiness and reduce physiological inertia at the onset of high-intensity exercise [15,43,47].

Interestingly, the physiological responses measured after the WIST did not directly reflect the observed performance outcomes. [La^−^] did not differ between conditions at 5 min post-exercise, but significant differences emerged at 10 min, with higher values in the dryland condition compared with the combined condition, and a moderate effect size was also observed relative to the in-water condition. Baseline [La^−^] values were similar across conditions, although the higher post-warm-up value observed in the dryland condition compared with the in-water condition may have partly contributed to the subsequent absolute values; however, it cannot explain the difference between the dryland and combined conditions at 10 min post-exercise. This delayed difference suggests that the primary variations between conditions may be related to recovery kinetics rather than the immediate metabolic response to exercise. In this context, the dryland condition may have induced a less favorable metabolic recovery profile, whereas the in-water and combined protocols may have facilitated a more efficient post-exercise metabolic state.

An additional explanation for these findings may relate to the distinct metabolic demands imposed by the warm-up protocols themselves. The dryland warm-up may have elicited a greater anaerobic contribution, potentially leading to higher glycolytic activation and greater lactate accumulation prior to the WIST. In contrast, the in-water component, particularly within the combined protocol, may have promoted a greater aerobic contribution and enhanced blood flow, which could have facilitated lactate clearance and contributed to the lower [La^−^] values observed during recovery. This interpretation is consistent with evidence indicating that prior exercise can modulate subsequent metabolic responses, including lactate production and clearance, as well as oxygen uptake kinetics [48]. In particular, warm-up-induced increases in muscle temperature, muscle perfusion, and oxygen delivery may enhance metabolite removal and improve the balance between lactate production and clearance, potentially contributing to more efficient recovery dynamics [12,49,50].

Regarding the HR, no significant differences were observed between conditions following the WIST. This may indicate that the test imposed a comparable cardiovascular stress across warm-up conditions. Although participants achieved greater distances after the in-water and combined protocols, the additional work performed may have compensated for any improvement in movement efficiency or physiological readiness, resulting in similar HR responses. Moreover, post-exercise HR is strongly influenced by autonomic reactivation and may be less sensitive than performance measures to the acute effects of different warm-up strategies [51,52,53]. Therefore, the lack of differences in HR does not necessarily indicate that the warm-up protocols induced identical preparatory responses, but rather that these differences were not reflected in the cardiovascular recovery measured after the WIST.

Tympanic temperature responses should be interpreted with caution in the present context. The non-existence of differences immediately after the WIST does not necessarily indicate that the thermal responses were identical between conditions. In aquatic exercise, the water environment strongly influences heat exchange, and immersion can attenuate or mask temperature responses measured immediately after exercise, particularly due to convective heat loss [15,34,43,51]. This may explain why the values at 1 min post-WIST were relatively low across conditions, while the 10 min values were higher and showed a tendency toward greater values in the combined condition. The literature consistently demonstrates that water immersion accelerates heat dissipation and alters thermoregulatory responses, particularly in the early recovery phase [47]. Moreover, the increase in muscle temperature induced by warm-up is known to be transient and can rapidly decline during inactivity or exposure to cooler environments, which may further contribute to the observed pattern [3,15,43,54].

Taken together, these findings suggest that the performance benefits of warm-up in water polo were more closely associated with task-specific preparation and immediate readiness than with marked differences in the physiological responses measured after the WIST. This is consistent with current evidence indicating that the ergogenic effects of warm-up are primarily mediated through acute neuromuscular, metabolic, and thermoregulatory adjustments that enhance initial performance capacity [12,43]. From a practical perspective, these results support the implementation of well-structured in-water warm-up routines, with or without a preceding dryland component, when the objective is to optimize short-term intermittent performance. Given that the combined protocol did not outperform the in-water condition, coaches should prioritize sport-specific aquatic preparation and should not assume that adding a dryland component will necessarily provide further benefits. The duration and overall physiological demand of the combined warm-up should also be considered.

Some limitations should be acknowledged. First, the sample size was relatively small. Although the randomized, counterbalanced, within-subject design reduced the influence of between-participant variability, the relatively small sample size may increase the risk of Type II errors and reduce the statistical power to detect small-to-moderate effects, thereby limiting the precision, robustness, and generalizability of the findings. Second, only male high-level water polo players were included, which limits generalization to female players and athletes from other competitive levels. The potential influence of playing position was also not considered, despite the distinct physiological and technical demands associated with different roles in water polo. Furthermore, the absence of a control condition without warm-up limits the interpretation of the absolute effectiveness of the tested protocols. The analysis of passive recovery following warm-up was limited to a 5 min passive recovery period. This duration may not accurately represent a full range of effects a warm-up can have. Finally, different transition times to the main activity can significantly influence the effectiveness of the warm-up. This limitation warrants further investigation in future studies involving water polo athletes. Future studies should also investigate whether similar patterns are observed in female players, different competitive levels, distinct playing positions, and under varying transition times between warm-up and task execution.

## 5. Conclusions

In conclusion, warm-up protocols including an in-water component were more effective than a dryland-only routine for optimizing intermittent performance in high-level water polo players. However, under the conditions examined in the present study, adding the tested dryland component to the in-water warm-up did not provide additional performance benefits. The findings reinforce the importance of specificity in warm-up design and suggest that the physiological state induced immediately after warm-up may help explain subsequent performance, even when post-exercise physiological responses are largely similar across conditions.

## Figures and Tables

**Table 1:** Dryland, In-water, and Combined protocols.

Phase	Dryland	In-Water	Combined
Activation	Arm rotations (forward/backward); chest and back mobility; shoulder activation; hip rotations 90/90′s	200 m mixed swimming (freestyle/backstroke); 4 × 25 m technique drills W/10 s rest (head-up swimming, breaststroke, butterfly variations) 4 × 25 m progressive intensity W/1 min rest	Dryland + In-water (same sequence)
Specific	Normal Squat Side to Side Squat Jumping Jacks Lunge Walk + Reverse Lunge with trunk rotation Lunge “Split” Plank Side Bridge Mountain climbers Burpees	1XEggbeater kicking (30 s on/30 s off); Technical drills; 1 vs. 1 drills; positional work; eggbeater variations; goalkeeper-specific drills 4X25 Drill (25 m—Eggbeater kicks; 25 m—Slide side to side with 1 breast kick; 25 m—Jump with one arm and with two arms; 25 m—Butterfly stroke with breaststroke kick W/10 s 1 vs.1: 1st (arms in shoulders) and face to face (horizontal position); 2nd pushes across colleague’s shoulder, with breaststroke kick/eggbeater; 3rd back-to-back; 4th Side by side (hand/arm hand on the colleague’s shoulder/back)—30 s on/30 s off each work Goalies: 1st “Walk” fingers touching cross bar; 2nd Steady position; 3rd Side to side high/low corner face the goal; 4th Lunging left/right high/low corner—30 s on/30 s off each work	
Final		Passing drills; steals; shooting drills (short/long passes, positional shooting): 1st Passing (Short/Long)—2 Rounds of 1′ W/15 s rest; 2nd Steals and “middle Steals”—2 Rounds of 1′ W/15 s rest; 3rd Shots (Specific positions; After a pass from the 2 m line; free—head shot; lob shot)—5′ (Rotation of initial and substitute players)	
Duration	~20 min	~20 min	~40 min

**Table 2:** Performance, physiological, and perceptual responses following the WIST under each warm-up condition.

	Dryland	In-Water	Combined
Distance (m)	226.5 ± 129.51	331.5 ± 151.24	358.5 ± 134.06
	(133.86, 319.14)	(223.32, 439.68)	(262.6, 454.4)
Average Speed (m/s)	1.38 ± 0.06	1.42 ± 0.05	1.43 ± 0.05
	(1.33, 1.42)	(1.38, 1.46)	(1.4, 1.47)
Temperature (°C; 1′)	35.82 ± 0.89	35.64 ± 0.41	35.54 ± 0.36
	(35.18, 36.46)	(35.35, 35.93)	(35.28, 35.79)
Temperature (°C; 10′)	36.80 ± 0.23	36.77 ± 0.29	36.96 ± 0.27
	(36.63, 36.97)	(36.56, 36.98)	(36.77, 37.15)
HR (beats/min; 1′)	133 ± 27.02	127.4 ± 20.27	126 ± 15.26
	(113.67, 152.33)	(112.9, 141.9)	(115.08, 136.92)
HR (beats/min; 10′)	96 ± 9.43	97.2 ± 9.81	89.6 ± 15.23
	(89.26, 102.74)	(90.18, 104.22)	(78.71, 100.49)
[La^−^] (mmol/L; 5′)	12.62 ± 4.41	11.86 ± 3.09	11.82 ± 3.01
	(9.46, 15.78)	(9.65, 14.07)	(9.67, 13.97)
[La^−^] (mmol/L; 10′)	12.46 ± 4.46	10.77 ± 2.86	8.66 ± 2.47
	(9.27, 15.65)	(8.73, 12.81)	(6.89, 10.43)
RPE (AU; 1′)	7.5 ± 0.85	7 ± 1.05	6.8 ± 1.4
	(6.89, 8.11)	(6.25, 7.75)	(5.8, 7.8)
RPE (AU; 10′)	3.5 ± 1.08	3.8 ± 0.92	3.4 ± 0.84
	(2.73, 4.27)	(3.14, 4.46)	(2.8, 4)

Values are presented as mean ± SD (95% confidence interval). Differences among warm-up conditions were assessed using one-way repeated-measures ANOVA, followed by LSD post hoc pairwise comparisons when appropriate. AU, arbitrary units; HR, heart rate; LSD, least significant difference; RPE, rating of perceived exertion; SD, standard deviation; WIST, Water Polo Intermittent Shuttle Test; [La^−^], blood lactate concentration.

**Table 3:** Pairwise comparisons of performance, physiological, and perceptual responses following the WIST.

	Dryland vs. In-Water	Dryland vs. Combined	In-Water vs. Combined
Distance (m)	ES = 2,	ES = 1.53,	ES = 0.29,
	*p* < 0.001 **	*p* = 0.001 **	*p* = 0.381
Average Speed (m/s)	ES = 1.9,	ES = 1.61,	ES = 0.35,
	*p* < 0.001 **	*p* = 0.001 **	*p* = 0.301
Temperature (°C; 1′)	ES = 0.24,	ES = 0.32,	ES = 0.19,
	*p* = 0.467	*p* = 0.343	*p* = 0.568
Temperature (°C; 10′)	ES = 0.08,	ES = 0.52,	ES = 0.57,
	*p* = 0.817	*p* = 0.133	*p* = 0.103
HR (beats/min; 1′)	ES = 0.23,	ES = 0.30,	ES = 0.10,
	*p* = 0.484	*p* = 0.301	*p* = 0.758
HR (beats/min; 10′)	ES = 0.10,	ES = 0.54,	ES = 0.65,
	*p* = 0.752	*p* = 0.125	*p* = 0.070
[La^−^] (mmol/L; 5′)	ES = 0.26,	ES = 0.17,	ES = 0.01,
	*p* = 0.429	*p* = 0.611	*p* = 0.969
[La^−^] (mmol/L; 10′)	ES = 0.59,	ES = 0.80,	ES = 0.58,
	*p* = 0.094	*p* = 0.033 *	*p* = 0.101
RPE (AU; 1′)	ES = 0.39,	ES = 0.6,	ES = 0.15,
	*p* = 0.244	*p* = 0.089	*p* = 0.642
RPE (AU; 10′)	ES = 0.22,	ES = 0.06,	ES = 0.37,
	*p* = 0.496	*p* = 0.847	*p* = 0.269

*p* values were obtained from LSD post hoc pairwise comparisons following one-way repeated-measures ANOVA. ES represents Hedges’ g.; ES, effect size; HR, heart rate; LSD, least significant difference; RPE, rating of perceived exertion; WIST, Water Polo Intermittent Shuttle Test; [La^−^], blood lactate concentration. * *p* ≤ 0.05; ** *p* < 0.01.

**Table 4:** Physiological and perceptual responses measured 5 min after each warm-up condition (Wup).

	Dryland	In-Water	Combined
Temperature. after warm-up	36.93 ± 0.4	36.61 ± 0.17	36.64 ± 0.46
	(36.64, 37.22)	(36.49, 36.73)	(36.31, 36.97)
HR after warm-up	99.2 ± 10.96	100.8 ± 7.73	92.5 ± 12.36
	(91.36, 107.04)	(95.27, 106.33)	(83.66, 101.34)
[La^−^] after warm-up	10 ± 3.83	7.18 ± 2.35	9.05 ± 2.83
	(7.26, 12.74)	(5.5, 8.86)	(7.03, 11.07)
RPE after warm-up	3.7 ± 0.67	4 ± 0.47	4.4 ± 0.84
	(3.22, 4.18)	(3.66, 4.34)	(3.8, 5)

Values are presented as mean ± SD (95% confidence interval). Differences among warm-up conditions were assessed using one-way repeated-measures ANOVA, followed by LSD post hoc pairwise comparisons when appropriate. HR, heart rate; LSD, least significant difference; RPE, rating of perceived exertion; SD, standard deviation; [La^−^], blood lactate concentration.

**Table 5:** Pairwise comparisons of physiological and perceptual responses measured 5 min after each warm-up condition.

	Dryland vs. In-Water	Dryland vs. Combined	In-Water vs. Combined
Temperature 5 min after warm-up	ES = 0.74,	ES = 0.51,	ES = 0.07,
	*p* = 0.044 *	*p* = 0.144	*p* = 0.819
HR 5 min after warm-up	ES = 0.13,	ES = 0.63,	ES = 0.78,
	*p* = 0.689	*p* = 0.077	*p* = 0.034 *
[La^−^] 5 min after warm-up-up	ES = 0.81,	ES = 0.23,	ES = 0.82,
	*p* = 0.031 *	*p* = 0.489	*p* = 0.030 *
RPE 5 min after warm-up-up	ES = 0.44,	ES = 0.6,	ES = 0.37,
	*p* = 0.193	*p* = 0.089	*p* = 0.269

*p* values were obtained from LSD post hoc pairwise comparisons following one-way repeated-measures ANOVA. ES represents Hedges’ g.; ES, effect size; HR, heart rate; LSD, least significant difference; RPE, rating of perceived exertion; [La^−^], blood lactate concentration. * *p* ≤ 0.05.

## Data Availability

The raw data supporting the conclusions of this article will be made available by the authors on request.

## Supplementary Materials

The following supporting information can be downloaded at: https://www.mdpi.com/article/10.3390/jfmk11030297/s1, Table S1: Baseline physiological and perceptual responses under each warm-up condition.

## Abbreviations

The following abbreviations are used in this manuscript:

CI	Confidence intervals
ES	Effect size
HR	Heart rate
LSD	Least significant difference
RPE	Rating of perceived exertion
SD	Standard deviation
WIST	Water Polo Intermittent Shuttle Test
ηp^2^	Partial eta squared
[La^−^]	Blood lactate concentration

## References

[1] Bishop D. (2003). Warm Up II: Performance Changes Following Active Warm Up and How to Structure the Warm Up. Sports Med..

[2] Kilduff L.P., Finn C.V., Baker J.S., Cook C.J., West D.J. (2013). Preconditioning Strategies to Enhance Physical Performance on the Day of Competition. Int. J. Sports Physiol. Perform..

[3] Olivier M.H., Gorman A.D., Connick M.J., Holmberg P.M., Desbrow J., Kelly V.G. (2025). The Water Polo Intermittent Shuttle Test in Women’s Water Polo Players. Eur. J. Sport Sci..

[4] Bishop D., Middleton G. (2013). Effects of Static Stretching Following a Dynamic Warm-up on Speed, Agility and Power. J. Hum. Sport Exerc..

[5] Febbraio M.A., Carey M.F., Snow R.J., Stathis C.G., Hargreaves M. (1996). Influence of Elevated Muscle Temperature on Metabolism during Intense, Dynamic Exercise. Am. J. Physiol.-Regul. Integr. Comp. Physiol..

[6] Andrade D., Henriquez-Olguin C., Beltran A., Ramirez M., Labarca C., Cornejo M., Alvarez C., Ramirez-Campillo R. (2015). Effects of General, Specific and Combined Warm-up on Explosive Muscular Performance. Biol. Sport.

[7] Gil M.H., Neiva H.P., Sousa A.C., Marques M.C., Marinho D.A. (2019). Current Approaches on Warming up for Sports Performance: A Critical Review. Strength Cond. J..

[8] McGowan C.J., Pyne D.B., Raglin J.S., Thompson K.G., Rattray B. (2016). Current Warm-Up Practices and Contemporary Issues Faced by Elite Swimming Coaches. J. Strength Cond. Res..

[9] Cuenca-Fernández F., Boullosa D., López-Belmonte Ó., Gay A., Ruiz-Navarro J.J., Arellano R. (2022). Swimming Warm-Up and Beyond: Dryland Protocols and Their Related Mechanisms—A Scoping Review. Sports Med.-Open.

[10] Papadimitriou K., Tsalis G., Loupos D. (2015). The Acute Effects of Active or Passive Stretching Exercises in Swimming as a Unique Way of Warm Up Before 50m High Intensity. Inq. Phys. Educ. Sport.

[11] West D.J., Russell M., Bracken R.M., Cook C.J., Giroud T., Kilduff L.P. (2015). Post-Warmup Strategies to Maintain Body Temperature and Physical Performance in Professional Rugby Union Players. J. Sports Sci..

[12] McGowan C.J., Pyne D.B., Thompson K.G., Rattray B. (2015). Warm-Up Strategies for Sport and Exercise: Mechanisms and Applications. Sports Med..

[13] Fradkin A.J., Zazryn T.R., Smoliga J.M. (2010). Effects of Warming-up on Physical Performance: A Systematic Review with Meta-Analysis. J. Strength Cond. Res..

[14] Papagiannis G., Karatrantou K., Batatolis C., Ioakimidis P., Gerodimos V. (2024). Individuality Affects the Efficiency of Basketball Pre-Game Warm-Up on Players’ Performance. Sports.

[15] Silva N., Travassos B., Gonçalves B., Brito J., Abade E. (2020). Pre-Match Warm-Up Dynamics and Workload in Elite Futsal. Front. Psychol..

[16] De Sire A., Demeco A., Marotta N., Moggio L., Palumbo A., Iona T., Ammendolia A. (2021). Anterior Cruciate Ligament Injury Prevention Exercises: Could a Neuromuscular Warm-Up Improve Muscle Pre-Activation before a Soccer Game? A Proof-of-Principle Study on Professional Football Players. Appl. Sci..

[17] Sannicandro I., Monacis D., Colella D. (2024). Effects of a Warm up Integrated with Core Stability Exercises on the Motor Abilities in Young Soccer Players. Pedagog. Phys. Cult. Sports.

[18] Thapa R., Clemente F., Moran J., Garcia-Pinillos F., Scanlan A.T., Ramirez-Campillo R. (2023). Warm-up Optimization in Amateur Male Soccer Players:A Comparison of Small-Sided Games and Traditional Warm-Uproutines on Physical Fitness Qualities. Biol. Sport.

[19] Chen C.-H., Chang C.-K., Tseng W.-C., Chiu C.-H., Dai X., Ye X. (2022). Acute Effects of Different Warm-up Protocols on Sports Performance in Elite Male Collegiate Handball Players. J. Strength Cond. Res..

[20] Steib S., Zahn P., Zu Eulenburg C., Pfeifer K., Zech A. (2016). Time-Dependent Postural Control Adaptations Following a Neuromuscular Warm-up in Female Handball Players: A Randomized Controlled Trial. BMC Sports Sci. Med. Rehabil..

[21] Fridvalszki M., Matlák J., Kovács B., Petridis L., Horváth D., Havanecz K., Dudás D., Langmár G., Rácz L. (2022). Reliability Study of a Functional Test for the Offensive Agility Performance in Water Polo. Int. J. Environ. Res. Public Health.

[22] Lupo C., Condello G., Capranica L., Tessitore A. (2014). Women’s Water Polo World Championships: Technical and Tactical Aspects of Winning and Losing Teams in Close and Unbalanced Games. J. Strength Cond. Res..

[23] Martin A.C., Smith M.L., Jones R.L. (2019). Physical and Physiological Attributes of Elite Water Polo Players Are Associated with Playing Position and Role in the Team. Int. J. Sports Sci. Coach..

[24] Platanou T., Geladas N. (2006). The Influence of Game Duration and Playing Position on Intensity of Exercise during Match-Play in Elite Water Polo Players. J. Sports Sci..

[25] Smith H.K. (1998). Applied Physiology of Water Polo. Sports Med..

[26] Hohmann A., Frase R. (1992). Analysis of Swimming Speed and Energy Metabolism in Competition Water Polo Games. Biomechanics and Medicine in Swimming. Swimming Science VI.

[27] Neiva H.P., Marques M.C., Barbosa T.M., Izquierdo M., Marinho D.A. (2014). Warm-Up and Performance in Competitive Swimming. Sports Med..

[28] Crowley E., Harrison A.J., Lyons M. (2018). Dry-Land Resistance Training Practices of Elite Swimming Strength and Conditioning Coaches. J. Strength Cond. Res..

[29] McKenzie M.R., McKean M.R., Doyle D.P., Hogarth L.W., Burkett B.J. (2022). Swimming Performance, Physiology, and Post-Activation Performance Enhancement Following Dryland Transition Phase Warmup: A Systematic Review. PLoS ONE.

[30] Mujika I., McFadden G., Hubbard M., Royal K., Hahn A. (2006). The Water-Polo Intermittent Shuttle Test: A Match-Fitness Test for Water-Polo Players. Int. J. Sports Physiol. Perform..

[31] West D.J., Dietzig B.M., Bracken R.M., Cunningham D.J., Crewther B.T., Cook C.J., Kilduff L.P. (2013). Influence of Post-Warm-up Recovery Time on Swim Performance in International Swimmers. J. Sci. Med. Sport.

[32] Balilionis G., Nepocatych S., Ellis C.M., Richardson M.T., Neggers Y.H., Bishop P.A. (2012). Effects of Different Types of Warm-Up on Swimming Performance, Reaction Time, and Dive Distance. J. Strength Cond. Res..

[33] Czelusniak O., Favreau E., Ives S.J. (2021). Effects of Warm-Up on Sprint Swimming Performance, Rating of Perceived Exertion, and Blood Lactate Concentration: A Systematic Review. J. Funct. Morphol. Kinesiol..

[34] Neiva H.P., Marques M.C., Barbosa T.M., Izquierdo M., Viana J.L., Teixeira A.M., Marinho D.A. (2015). The Effects of Different Warm-up Volumes on the 100-m Swimming Performance. J. Strength Cond. Res..

[35] McKay A.K.A., Stellingwerff T., Smith E.S., Martin D.T., Mujika I., Goosey-Tolfrey V.L., Sheppard J., Burke L.M. (2022). Defining Training and Performance Caliber: A Participant Classification Framework. Int. J. Sports Physiol. Perform..

[36] Ferragut C., Vila H., Abraldes J.A., Argudo F., Rodriguez N., Alcaraz P.E. (2011). Relationship among Maximal Grip, Throwing Velocity and Anthropometric Parameters in Elite Water Polo Players. J. Sports Med. Phys. Fit..

[37] Botonis P.G., Arsoniadis G.G., Smilios I., Toubekis A.G. (2025). In-Season Training Load Variation—Heart Rate Recovery, Perceived Recovery Status, and Performance in Elite Male Water Polo Players: A Pilot Study. Sports Health Multidiscip. Approach.

[38] Botonis P.G., Toubekis A.G., Platanou T.I. (2016). Concurrent Strength and Interval Endurance Training in Elite Water Polo Players. J. Strength Cond. Res..

[39] Takahashi J., Aoki J. (1998). The Effects of Recovery Mode on Blood Lactate Removal During Intervals in the Water-Polo Games. Jpn. J. Phys. Fit. Sports Med..

[40] Papadimitriou K., Kabasakalis A., Papadopoulos A., Mavridis G., Tsalis G. (2023). Comparison of Ultra-Short Race Pace and High-Intensity Interval Training in Age Group Competitive Swimmers. Sports.

[41] Nimah M.M., Bshesh K., Callahan J.D., Jacobs B.R. (2006). Infrared Tympanic Thermometry in Comparison with Other Temperature Measurement Techniques in Febrile Children. Pediatr. Crit. Care Med..

[42] Borg G. (1998). Borg’s Perceived Exertion and Pain Scales.

[43] Silva L.M., Neiva H.P., Marques M.C., Izquierdo M., Marinho D.A. (2018). Effects of Warm-Up, Post-Warm-Up, and Re-Warm-Up Strategies on Explosive Efforts in Team Sports: A Systematic Review. Sports Med..

[44] Goulding R.P., Burnley M., Wüst R.C.I. (2023). How Priming Exercise Affects Oxygen Uptake Kinetics: From Underpinning Mechanisms to Endurance Performance. Sports Med..

[45] Kawai E., Tsunokawa T., Sakaue H., Takagi H. (2023). Propulsive Forces on Water Polo Players’ Feet from Eggbeater Kicking Estimated by Pressure Distribution Analysis. Sports Biomech..

[46] De Sáez Villarreal E., Suarez-Arrones L., Requena B., Haff G.G., Ramos Veliz R. (2015). Enhancing Performance in Professional Water Polo Players. J. Strength Cond. Res..

[47] Neiva H.P., Marques M.C., Barbosa T.M., Izquierdo M., Viana J.L., Marinho D.A. (2017). Effects of 10min vs. 20min Passive Rest after Warm-up on 100m Freestyle Time-Trial Performance: A Randomized Crossover Study. J. Sci. Med. Sport.

[48] Burnley M., Doust J.H., Jones A.M. (2005). Effects of Prior Heavy Exercise on Metabolism and Performance during Severe Exercise. Eur. J. Appl. Physiol..

[49] Huang T., Liang Z., Wang K., Miao X., Zheng L. (2025). Novel Insights into Athlete Physical Recovery Concerning Lactate Metabolism, Lactate Clearance and Fatigue Monitoring: A Comprehensive Review. Front. Physiol..

[50] Raccuglia M., Lloyd A., Filingeri D., Faulkner S.H., Hodder S., Havenith G. (2016). Post-Warm-up Muscle Temperature Maintenance: Blood Flow Contribution and External Heating Optimisation. Eur. J. Appl. Physiol..

[51] Dupuy A., Birat A., Maurelli O., Garnier Y.M., Blazevich A.J., Rance M., Ratel S. (2022). Post-Exercise Heart Rate Recovery and Parasympathetic Reactivation Are Comparable between Prepubertal Boys and Well-Trained Adult Male Endurance Athletes. Eur. J. Appl. Physiol..

[52] Qiao J., Rosbrook P., Sweet D.K., Pryor R.R., Hostler D., Looney D., Pryor J.L. (2025). Does a Priming Warm-up Influence the Incidence of V˙O2pl during a Ramp Test and Verification Phase?. PLoS ONE.

[53] Fornasiero A., Savoldelli A., Skafidas S., Stella F., Bortolan L., Boccia G., Zignoli A., Schena F., Mourot L., Pellegrini B. (2018). Delayed Parasympathetic Reactivation and Sympathetic Withdrawal Following Maximal Cardiopulmonary Exercise Testing (CPET) in Hypoxia. Eur. J. Appl. Physiol..

[54] Yamashita Y., Umemura Y. (2024). Effect of High-Intensity with Short-Duration Re–Warm-Up on Subsequent Performance in a Cold Environment. J. Strength Cond. Res..

