# Supplementary material for: Effects of Different Warm-Up Strategies on Intermittent Performance and Physiological Responses in High-Level Water Polo Players
Source: J Funct Morphol Kinesiol. 2026 Jul 28;11(3):297. doi: 10.3390/jfmk11030297 (PMC13511443; doi:10.3390/jfmk11030297)
Supplement: Supplementary file 1 [file jfmk-11-00297-s001.zip › jfmk-4391810-supplementary.pdf]

Journal of
Functional Morphology and Kinesiology

Table S1. Baseline physiological and perceptual responses under each warm-up condition.

	Dryland	In-water	Combined	F	*p*	ηp²
**Temperature**	36.68 ± 0.25	36.78 ± 0.26	36.58 ± 0.21	1.68	0.215	0.157
**HR**	63.00 ± 5.83	63.20 ± 6.12	63.10 ± 3.60	0.00	0.995	0.001
**[La⁻]**	1.64 ± 0.15	1.64 ± 0.16	1.66 ± 0.13	0.05	0.952	0.005
**RPE**	1.20 ± 0.42	1.30 ± 0.48	1.10 ± 0.32	0.73	0.496	0.075

Values are presented as mean ± SD. Differences among warm-up conditions were assessed using one-way repeated-measures ANOVA; F, ANOVA test statistic; HR, heart rate; RPE, rating of perceived exertion; SD, standard deviation; $[La^-]$, blood lactate concentration; ηp^2, partial eta squared. Statistical significance was set at $p \leq 0.05$.
